# Synchronous tonsillar tumors with differing histopathology: A case report and review of the literature

**DOI:** 10.1002/cnr2.1615

**Published:** 2022-03-14

**Authors:** Nicholas A. Rossi, Devin N. Reddy, Jordan W. Rawl, Jianli Dong, Suimin Qiu, Cecilia G. Clement, Vicente A. Resto, Rohan Joshi, Brian McKinnon, Orly Coblens

**Affiliations:** ^1^ Department of Otolaryngology University of Texas Medical Branch Galveston Texas USA; ^2^ School of Medicine University of Texas Medical Branch Galveston Texas USA; ^3^ Department of Pathology University of Texas Medical Branch Galveston Texas USA

## Abstract

**Background:**

Tonsillar squamous cell carcinoma (TSCC) due to human papillomavirus (HPV) infection has seen a dramatic increase in recent years. Bilateral tonsillar squamous cell carcinoma (biTSCC) has a much lower incidence than unilateral TSCC and three main hypotheses of biTSCC pathogenesis prevail: field carcinogenesis, single‐clone, and multiple HPV infections.

**Case:**

A 49‐year‐old Male with a remote history of chewing tobacco presented with symptoms of spitting up tissue and occasional hemoptysis. Physical exam showed a sole left tonsillar mass which was confirmed to be TSCC on biopsy. The patient's computed tomographic (CT) scan was consistent with this finding; however, positron emission tomography (PET) scan indicated a second tumor in the contralateral right tonsil. Surgical resection of both masses and selective neck dissection was performed, and the specimens were sent for further pathological analysis. No complications of surgery were noted and the final diagnosis of synchronous biTSCC was made. The tumors were a T2N0M0 left poorly differentiated TSCC (p16+, EGFR+, bcl2+) with basaloid features, and a T1N0M0 right well to moderately differentiated TSCC (p16+, EGFR+, bcl2−).

**Conclusion:**

Our present case was notable for differing tumor pathology and karyotype analysis between the right and left masses, directly supporting the multiple HPV infections hypothesis of biTSCC pathogenesis. Further genetic characterization of tonsillar tumors is needed to better characterize TSCC and best guide medical/surgical therapy.

## 1 INTRODUCTION

Tonsillar squamous cell carcinoma (TSCC) is the most common oropharyngeal cancer and the third‐most common head and neck malignancy.^1^, ^2^ Despite a decline in tobacco use in recent decades, incidence rates of tonsillar carcinoma are increasing secondary to a growing prevalence of oropharyngeal human papillomavirus (HPV) infection.^3^, ^4^

TSCC is the most common of all oropharyngeal carcinomas, occurring at a rate of about 1.50 per 100 000 individuals without a history of tonsillectomy.^1^, ^2^, ^3^ Primary cancer of the tonsils is usually squamous, with one study citing a frequency of 85%.^5^ The remainder of cases are represented by lymphomas (12%) and less common subtypes such as basaloid squamous cell carcinoma (SCC), verrucous carcinoma, lymphoepithelioma, spindle cell carcinoma, adenosquamous carcinoma, acantholytic SCC, papillary SCC, and nuclear protein in testis midline carcinoma.^2^, ^4^, ^6^

The tonsils are the most common site of HPV+ oropharyngeal squamous cell carcinoma; Pinatti et al. hypothesized that the tonsils act as a reservoir for HPV, and the thin epithelium may be more susceptible to infection.^7^ Head and neck cancers have a 2.7%–4% probability of developing a synchronous primary tumor, for which there are three main hypotheses of origin^8^, ^9^, ^10^:

1. *Field carcinogenesis*: extended exposure to carcinogens (e.g., alcohol, tobacco, HPV) leads to separate metaplastic and dysplastic changes in epithelial cells at different sites.
2. *Single‐clone*: a single primary lesion gives off clonally related tumors which migrate to other locations and may present as synchronous or metachronous secondary primary malignancies.
3. *Multiple HPV infections*: multiple HPV infections in the oropharyngeal epithelium make it possible for independent tumors to arise at different locations.

Compared to unilateral disease, bilateral primary TSCC is less common, occurring at an incidence cited between 1% and 10%.^8^, ^9^, ^10^, ^11^, ^12^ The true incidence of synchronous TSCC is unknown, which may be attributed to a history of underreporting or underdiagnosing—only 38 cases have been reported to date.^10^, ^13^, ^14^

Treatment of head and neck squamous cell carcinoma (HNSCC) currently lacks the molecular characterization that is available for other types of malignancies such as breast and lung cancers.^15^ In our case, we present karyotype analysis of two tonsillar cancer cell lines with specific loci of genetic change.

## 2 CASE REPORT

A 49‐year‐old‐male with a remote history of chewing tobacco use presented to clinic with an 8‐month history of spitting up tissue and occasional hemoptysis. He denied odynophagia, dysphagia, otalgia, and weight loss. On examination, a left tonsil mass was noted to be extending to the midline and was firm to palpation. The base of tongue was soft and without visible lesions; no lesions or deformity were noted on the right tonsil. Biopsies were obtained in clinic of the left tonsillar mass and resulted squamous cell carcinoma.

**FIGURE 1:**
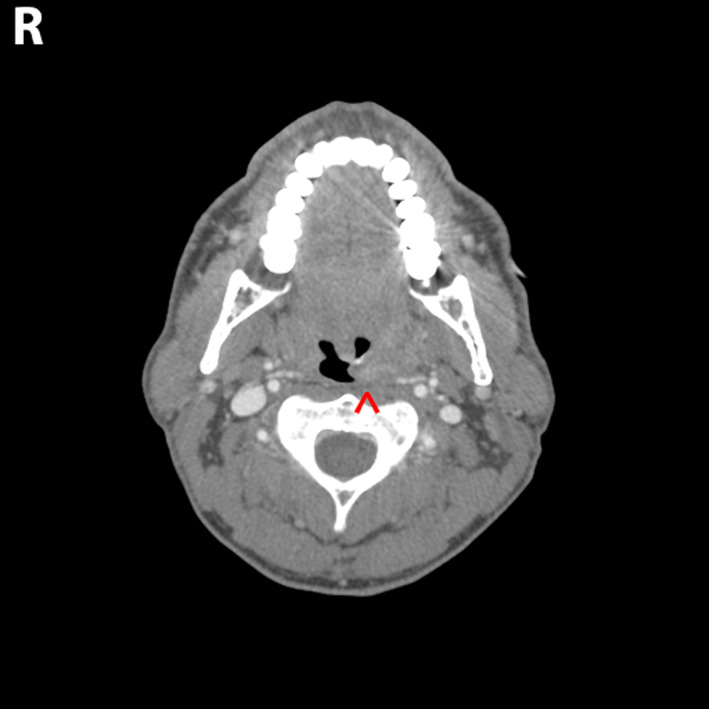
Computed tomographic (CT) neck with contrast. Axial view showing left palatine tonsillar mass extending to midline measuring 2 × 72.5 × 2.7 cm (red arrow)

**FIGURE 2:**
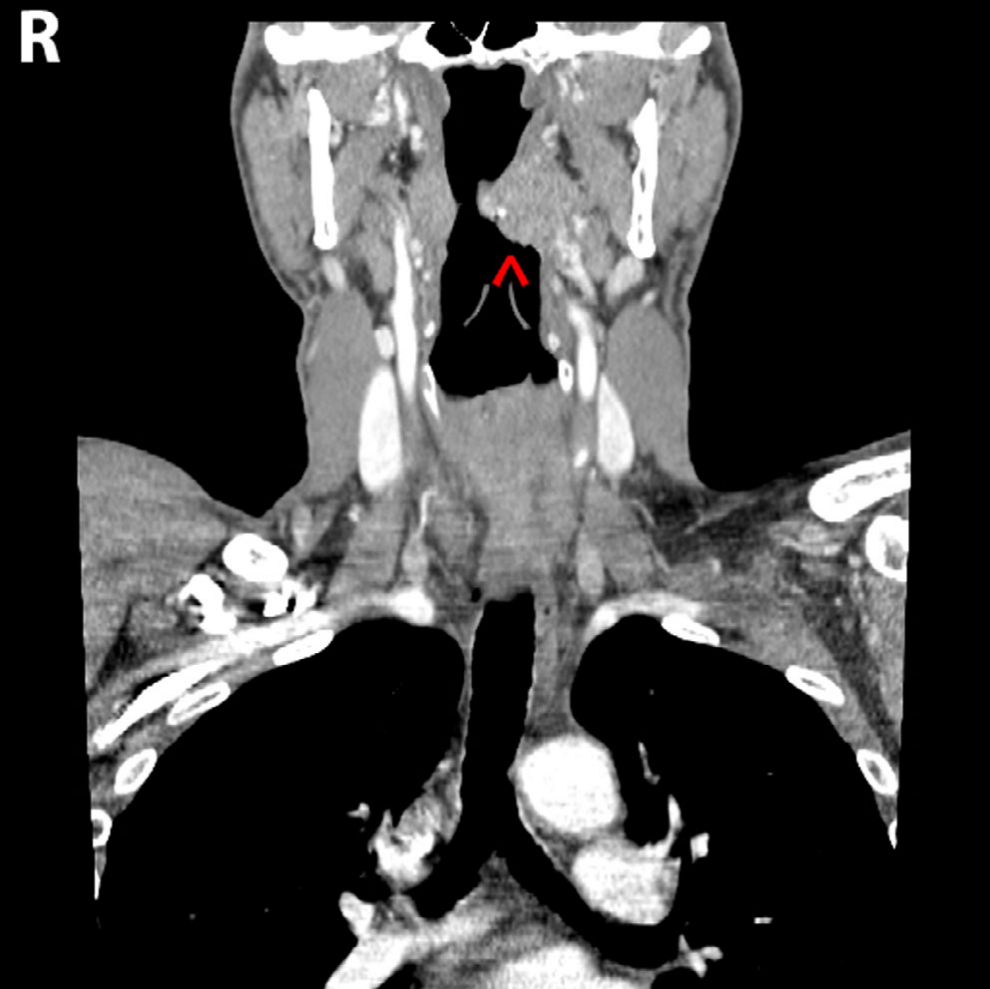
Computed tomographic (CT) neck with contrast. Coronal view showing the exophytic mass (red arrow) extending medially towards the uvula within the oropharynx

**FIGURE 3:**
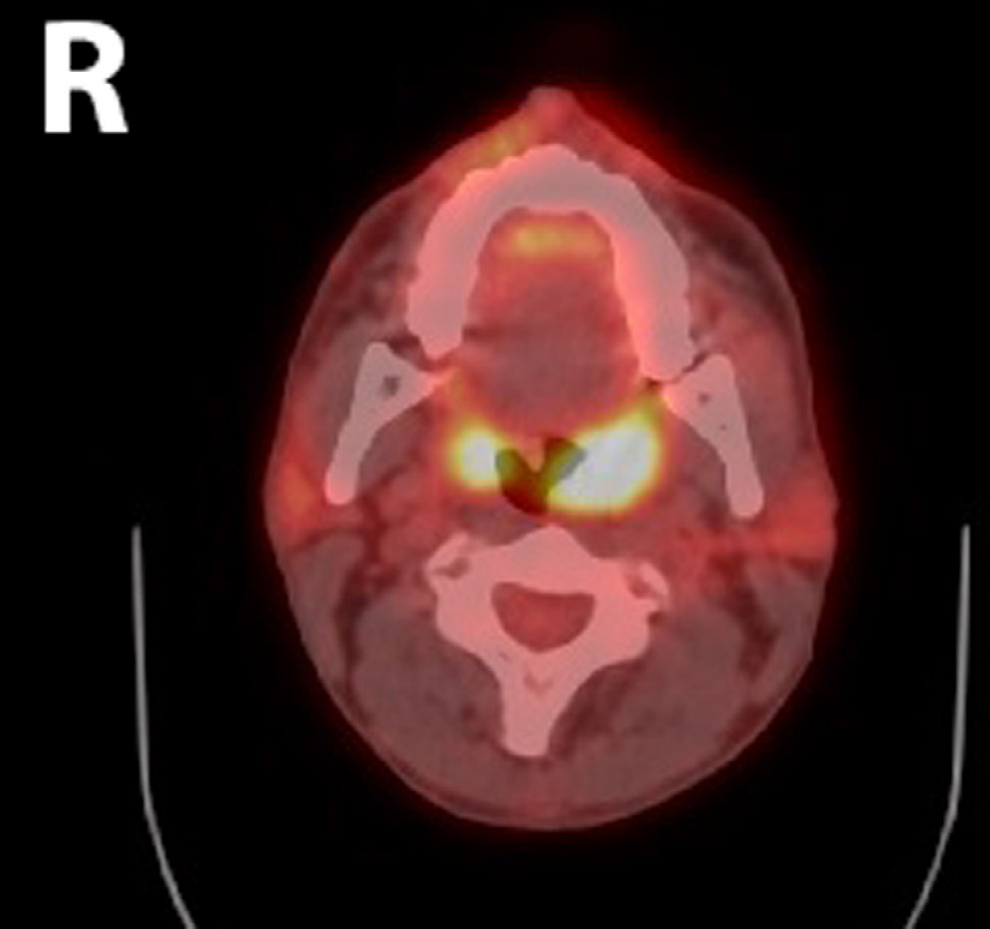
Whole body fluorodeoxyglucose (FDG)‐PET/computed tomographic (CT). Axial view showing increased radiotracer uptake in both the left and right palatine tonsils with SUV of 8.2 and 6.6, respectively

**FIGURE 4:**
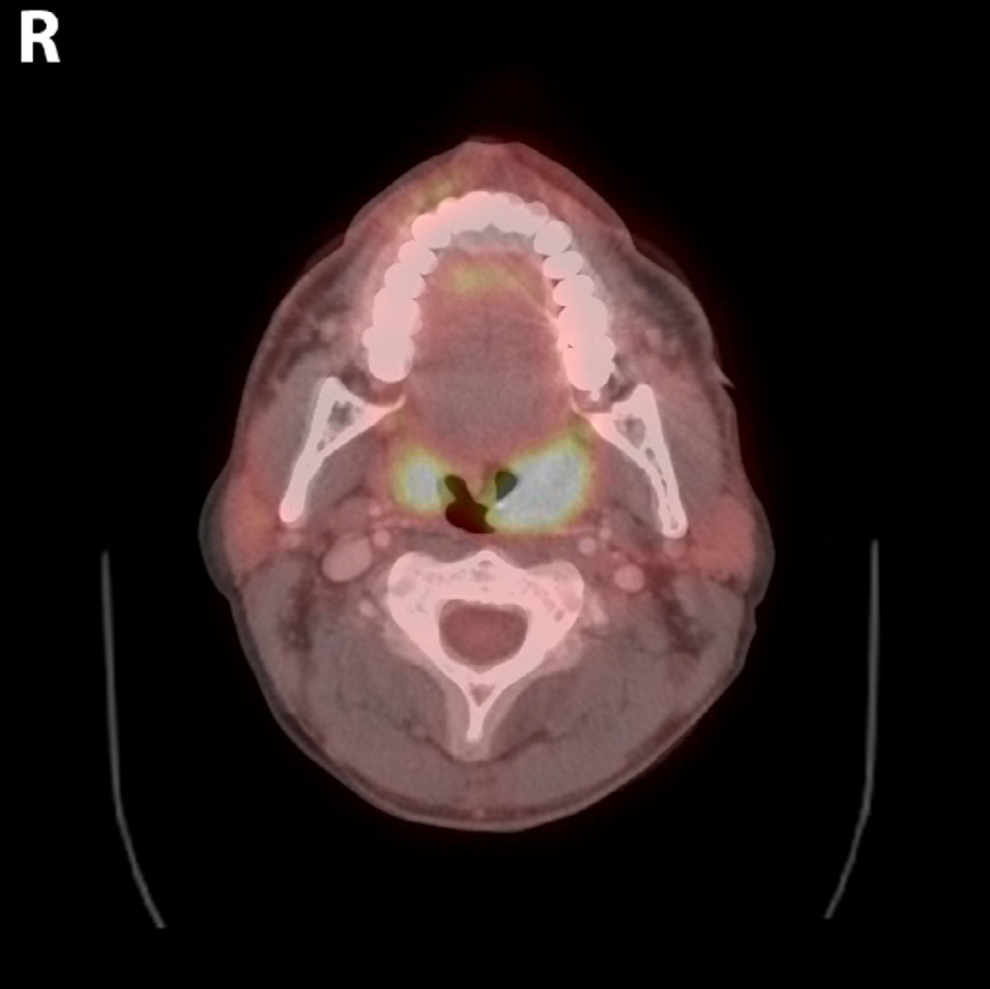
Axial view of whole body FDG‐PET/computed tomographic (CT) layered at 55% opacity over CT scan, allowing for improved visualization of the tumor margins in the oropharynx

A contrasted computed tomographic (CT) scan of the neck demonstrated a 2.0 × 2.5 × 2.7 cm left palatine tonsillar mass (Figures 1 and 2). No pathologically enlarged lymph nodes were noted. A positron emission tomography (PET) scan demonstrated increased radiotracer uptake in the left palatine tonsil with a standardized uptake value (SUV) of 8.2, along with the left nasal cavity with an SUV of 2.9 with mucosal thickening. Interestingly, the right palatine tonsil also demonstrated increased uptake with an SUV of 6.6 but no underlying soft tissue abnormality was detected on the CT scan nor on physical exam (Figures 3 and 4). Neither hypermetabolic lymph nodes nor lung nodules were detected.

**FIGURE 5:**
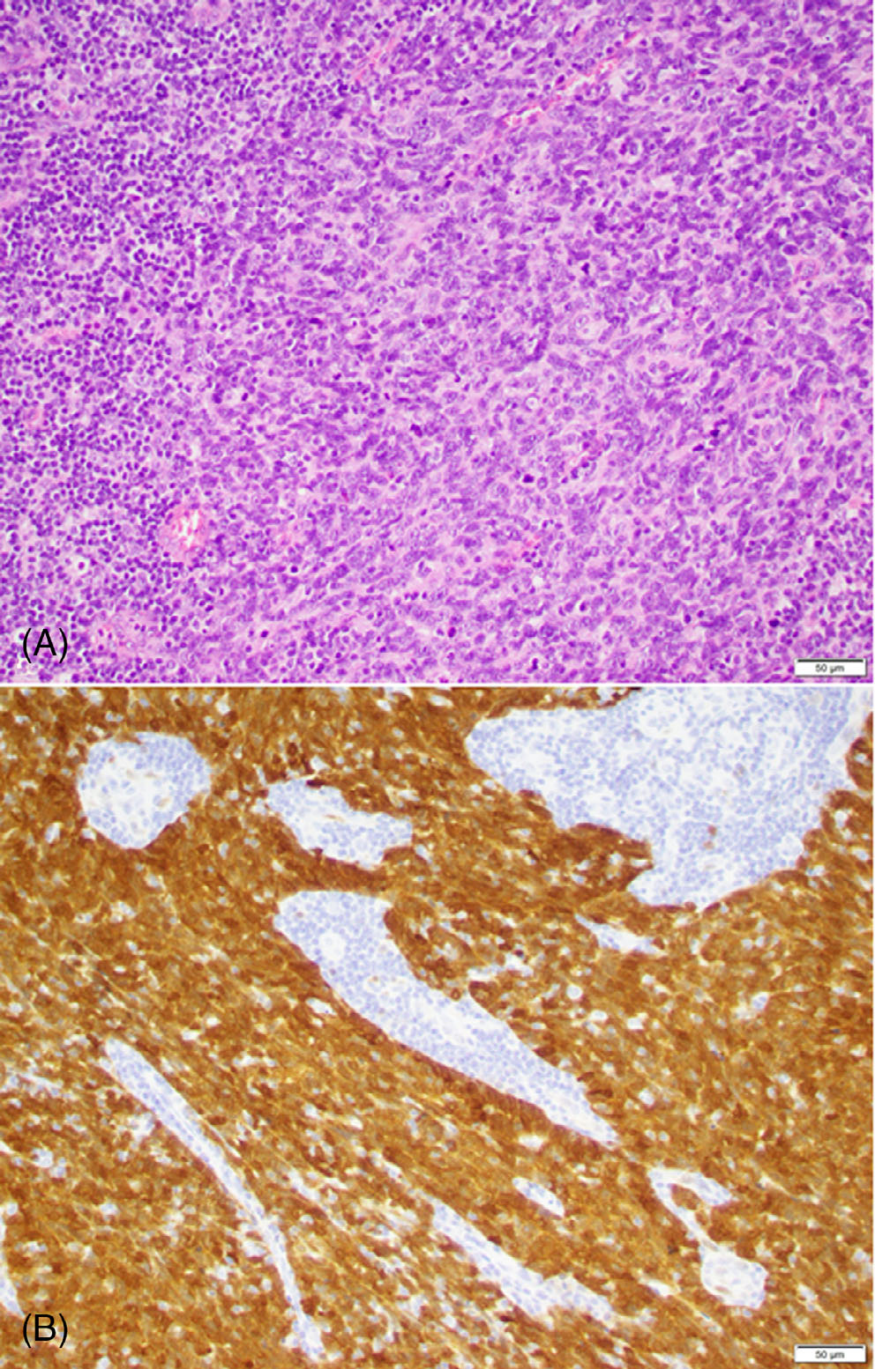
Both tumors are non‐keratinizing squamous cell carcinoma with similar morphology (A) and diffusely positive for p16 immunohistochemical stains (B), as demonstrated from the left tonsil specimen

One month later, the patient underwent a robotic right tonsillectomy, radical left tonsillectomy, and then pharyngoplasty with staged bilateral levels II–IV selective neck dissections 1 week later. The final diagnosis was synchronous TSCC including a T2N0M0 left poorly‐differentiated TSCC (p16+, EGFR+, bcl2+; Figure 5) with basaloid features and a T1N0M0 right well to moderately‐differentiated TSCC (p16+, EGFR+, bcl2−). Both tumors were HPV genotype 16. Head and neck tumor board analysis of the case supported close observation and no adjuvant therapy.

## 3 DISCUSSION

Tonsillar squamous cell carcinoma may present as oropharyngeal bleeding, dysphagia, non‐healing ulcer, hemoptysis, persistent halitosis, tonsillar asymmetry, otalgia, changes in articulation, or weight loss.^1^, ^2^, ^4^ The tonsils' rich lymphatic and vascular supply provides a favorable environment for early metastasis. Invasive disease usually presents as a neck mass, which may be cystic.^6^ The incidence of cervical lymph node metastases at the time of diagnosis has been cited at 39%–83%.^16^ Trismus, if detected, indicates a higher TNM grade and therefore a poor prognosis that suggests lymphatic spread of the tumor.^2^ The differential diagnosis of tonsillar malignancy includes tonsillitis, aphthous ulcer, chancre, oral candidiasis, leukoplakia, benign oropharyngeal lesion, or upper respiratory manifestation of systemic or rheumatic disease.^4^

Although TSCC has classically been associated with older males with a history of smoking and alcohol use, HPV's increasing role in carcinogenesis has been well documented in recent years.^3^, ^4^ Fakhry et al. reported 77% of tonsillar carcinomas in Denmark from 2000 to 2010 were HPV‐related.^3^ Workup and diagnosis of TSCC is achieved through a combination of detailed history, thorough physical examination, imaging, and histopathological analysis. Fine‐needle aspiration ought to be performed for suspicious lymphadenopathy.^2^ Once the diagnosis has been made, panendoscopy should be performed under general anesthesia to assess for feasibility of surgical resection with or without transoral robotic surgery (TORS) and to evaluate for the possibility of any second primary or additional mucosal changes that may indicate early premalignant lesions. While many authors support the use of PET/CT, one major drawback to consider is its high false‐positive rate due to infections, foreign bodies, healing, or other local processes destructive to the surrounding tissue.^17^ Shimizu et al. reported detecting bilateral TSCC using transoral endoscopic examination with narrow‐band imaging; however, further evaluation of this method is warranted before widespread adoption of this practice.^18^

Optimal treatment for TSCC remains controversial and under continued revision. Options include surgical excision with or without chemoradiation, depending on the stage of the tumor. Given the more favorable prognosis of HPV+ tonsillar tumors compared to their HPV− negative counterparts, de‐escalation protocols utilizing 60 Gy radiation as opposed to the usual 70 Gy have become more common practice.^19^ Bilateral radiation therapy for HPV+ tonsillar cancer may result in significantly higher healthcare resource utilization as compared to unilateral radiation therapy and should only be considered if there is a strong medical indication.^20^ Additionally, bilateral radiation therapy was associated with poorer quality of life and increased toxicity complications.^21^ Unilateral radiation therapy can be best considered for patients with a low risk of contralateral neck failure.

The topic of surgical resection of tonsillar carcinomas has been hotly debated in the past several years, primarily concerning bilateral or unilateral tonsillectomy in the case of a known or suspected unilateral tonsillar carcinoma. Dziegielewski et al. and Rokkiaer et al. recommend bilateral tonsillectomy in all patients with suspected or proven tonsillar carcinoma, unilateral or bilateral, and carcinoma of unknown primary.^10^, ^11^ On the contrary, Parhar et al. and McMillan et al. suggest unilateral wide‐field tonsillectomy in the case of unilateral HPV+ TSCC, unless high‐risk features are present.^8^, ^22^ The difference in these suggestions center on differing assessment of surgical risk, field cancerization, development of further primary tumors, functional patient outcomes, quality of life, and other complications of care.

In this case report, we present an example of bilateral synchronous primary tonsillar carcinoma of differing histopathology. To our knowledge, only four cases of bilateral tonsillar tumors with differing histopathology have been reported in the literature to date (Table 1).^2^, ^13^, ^14^, ^23^, ^24^, ^25^ It is unknown if this figure (10.5%, or 4/38 case reports) is significant—high or low—and may be a byproduct of differing histopathology reporting/descriptive practices in the literature. Three of these four cases were reportedly p16+, as in our case. This is the first case to report either epidermal growth factor receptor (EGFR)‐positivity or bcl2‐positivity in the presence of bilateral tonsillar tumors.

**TABLE 1:** Previously reported cases of synchronous bilateral TSCC with differing histopathology

Author	Year	Cases	HPV	p16	EGFR	bcl2	Primary diagnosis	Pathology
Koch	2001	1	−	n/a	n/a	n/a	CUP	*Right*: carcinoma in situ *Left*: T1 primary lesion
McGovern	2010	1	+	+	n/a	n/a	CUP	*Right*: moderate to poorly differentiated SCC *Left*: moderately differentiated SCC
Moualed	2011	1	−	+	n/a	n/a	Unilateral carcinoma	*Right*: invasive primary SCC *Left*: primary basaloid SCC
Roeser	2011	1	+	+	n/a	n/a	CUP	*Right*: invasive moderately to poorly differentiated keratinizing squamous cell carcinoma with marked desmoplasia *Left*: moderately to poorly differentiated non‐keratinizing squamous cell carcinoma associated with only a focal desmoplastic stromal reaction

Abbreviations: CUP, carcinoma of unknown primary; HPV, human papillomavirus; SCC, squamous cell carcinoma; TSCC, tonsillar squamous cell carcinoma.

**FIGURE 6:**
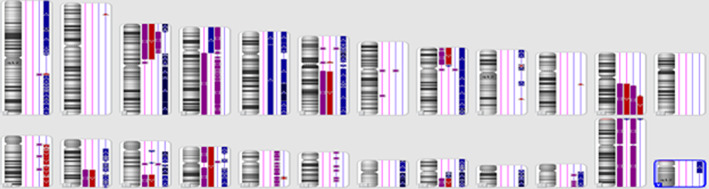
Single nucleotide polymorphism (SNP) chromosomal microarray analysis of both right and left tonsillar DNA (right tonsil = blue lines, left tonsil = pink lines). In the right tonsil, DNA copy number changes were detected including gains in chromosomes 1, 3, 5, 6, 8, 9, 14, 15, 16, 19, 20, 21, 22, and Y (blue bars), losses in chromosomes 1, 11, 13, and 17 (red bars), and copy‐neutral loss of heterozygosity (LOH) in chromosomes 3, 4, 11, 17, 18, and 22 (purple bars). In the left tonsil, DNA copy number changes were detected including gains in chromosomes 4, 5, 8, 15, 20, and Y; losses in chromosomes 3, 6, 8, 11, 14, 15, 16, and 20, and LOH in chromosome 4

In addition to differing histopathologic features, each of the two tonsillar specimens in our case were karyotyped and found to contain unique chromosomal copy number gains and losses, even though they were the same HPV genotype (type 16; Figure 6). These karyotyping differences are unlikely to be derived from the same viral lineage, suggesting that this patient's bilateral disease resulted from two distinct neoplastic events. This finding provides moderately strong support for the ‘Multiple HPV Infections’ hypothesis for bilateral tonsillar tumor origin. The ‘Field Carcinogenesis’ hypothesis may have had a more secondary/minor role in the pathogenesis of this patient's disease; however, the ‘Single‐Clone’ hypothesis is much less likely to have played a significant role. Saber et al. similarly reported two cases of biTSCC in which the tumors were HPV16+/HPV33+ and HPV16+/HPV35+, however no differences in the histopathology were noted in either case.^9^

In contrast to the two distinct karyotypes seen in our patient, Pinatti et al. suggested a common origin of a tumor in their case report on an HPV+ p16+ bilateral TSCC.^7^ Through analysis of HPV integration events, the authors ultimately supported single‐clone hypothesis of bilateral tumor development, although, again, no differing histopathological specimens were noted between tonsils. More generally, all three hypotheses of bilateral tonsillar carcinoma origin have validity and the weight of their respective contribution varies on a case‐by‐case basis.

In the future, we hope to be able to associate specific genetic loci to these chromosomal changes and determine which of these loci are clinically significant, with the end goal of developing targeted molecular therapy. In one study, Seiwert et al. sequenced 120 head and neck tumor specimens for hundreds of mutations.^15^ Their work revealed targetable genomic alterations in *FGFR1*, *DDR2*, *EGFR*, *FGFR2/3*, *EPHA2*, and *PIK3CA*. However, Cetuximab—an antibody directed against the *EGFR* receptor—is the only approved treatment for HNSCC that is directed against a specific molecular target. Given the increasing incidence of HPV‐related oropharyngeal cancers, this case highlights the need for fastidious work up of patients with unilateral tumors for disease on the contralateral side. Our hope is that further research will identify HPV‐associated genetic changes, with the goal of individualized pharmacologic therapy.

## CONFLICT OF INTEREST

The authors declared that they have no conflict of interest to this work.

## AUTHOR CONTRIBUTIONS

**Nicholas A. Rossi**: Conceptualization (equal); writing – original draft (equal); writing – review and editing (equal). **Devin N. Reddy**: Conceptualization (equal); writing – original draft (equal); writing – review and editing (equal). **Jordan W. Rawl:** Conceptualization (equal); writing – original draft (equal); writing – review and editing (equal). **Jianli Dong:** Investigation (equal); supervision (equal); writing – review and editing (equal). **Suimin Qiu:** Data curation (equal); investigation (equal); supervision (equal); writing – review and editing (equal). **Cecilia G. Clement:** Data curation (equal); project administration (equal); writing – review and editing (equal). **Vincent A. Resto:** Conceptualization (equal); supervision (equal); writing – review and editing (equal). **Rohan Joshi:** Conceptualization (equal); data curation (equal); investigation (equal); supervision (equal); writing – review and editing (equal). **Brian McKinnon:** Investigation (equal); project administration (equal); resources (equal); supervision (equal); writing – review and editing (equal). **Orly Coblens:** Conceptualization (equal); investigation (equal); resources (equal); supervision (equal); writing – review and editing (equal).

## ETHICS STATEMENT

I, Nicholas Armando Rossi, assure that for the manuscript herein, this material is the authors' own original work and has not been previously published elsewhere, although it was presented as a poster at the 2019 AAO‐HNSF Annual Meeting in Atlanta, GA. The paper is not currently being considered for publication elsewhere, and the content reflects the authors' own case in a truthful and complete manner. Meaningful contributions of co‐authors have been completely acknowledged. All sources used are properly disclosed. Finally, all authors have been actively involved in substantial work leading to completion of the paper and assume responsibility for its content.
